# Supplementary figures and images for: Loss of prion protein control of glucose metabolism promotes neurodegeneration in model of prion diseases
Source: PLoS Pathog. 2021 Oct 5;17(10):e1009991. doi: 10.1371/journal.ppat.1009991 (PMC8519435; doi:10.1371/journal.ppat.1009991)

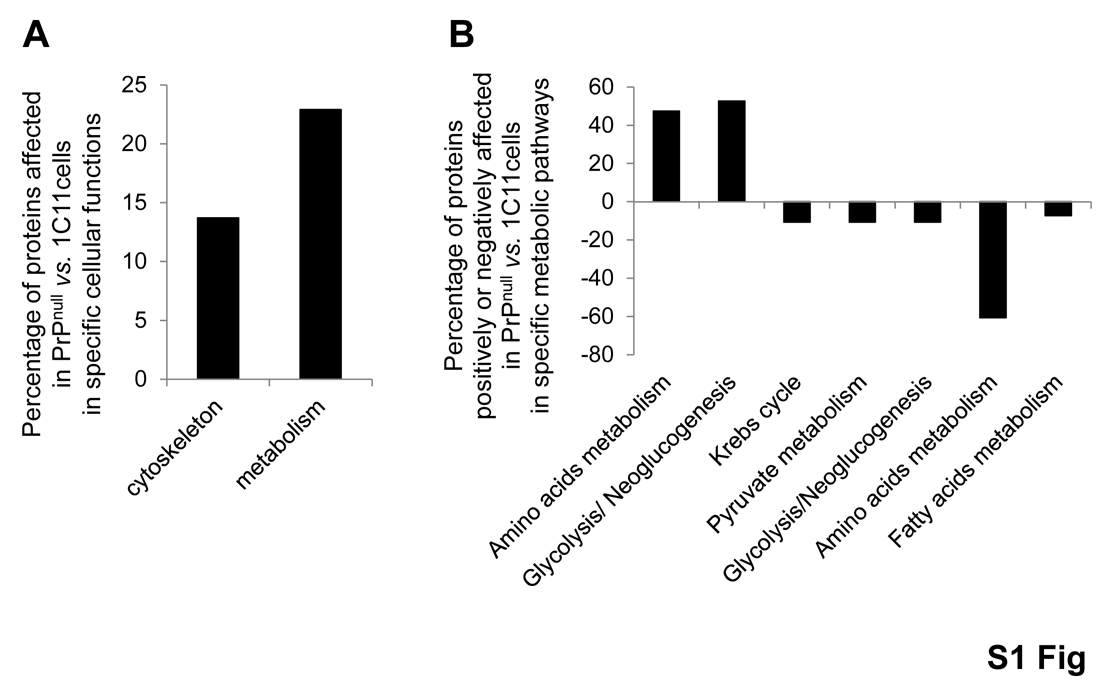

Supplement: S1 Fig — (A) Cellular functions and (B) metabolic pathways mainly affected by the absence of PrPC in 1C11 neuronal stem cells. Data are presented as the percentage of proteins involved in a specific cell function or metabolic pathway, whose expression is affected by the silencing of PrPC. (TIF) [file ppat.1009991.s001.tif]

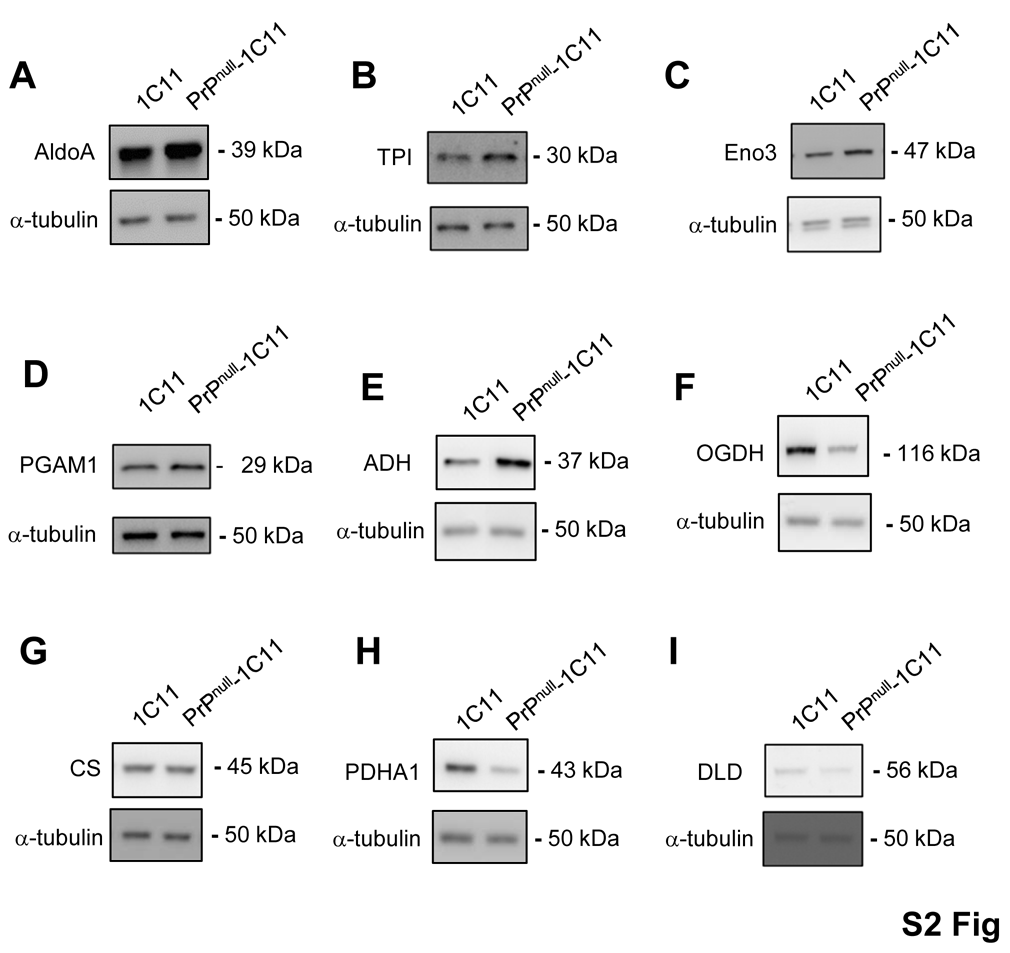

Supplement: S2 Fig — Representative Western blots of AldoA (A), TPI (B), Eno3 (C), PGAM1 (D), ADH (E), OGDH (F), CS (G), PDHA1 (H), and DLD (I) expression in 1C11 and PrPnull-1C11 cells. α-tubulin was used for normalization. (TIF) [file ppat.1009991.s002.tif]

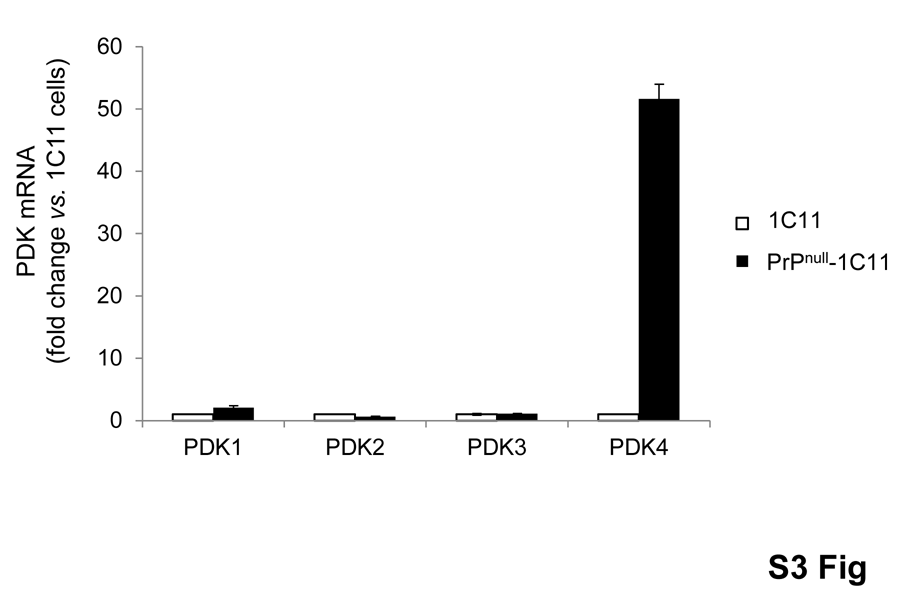

Supplement: S3 Fig — Relative mRNA levels of PDK1, PDK2, PDK3 and PDK4 between 1C11 and PrPnull-1C11 cells as assessed by RT-qPCR (n = 3). (TIF) [file ppat.1009991.s003.tif]

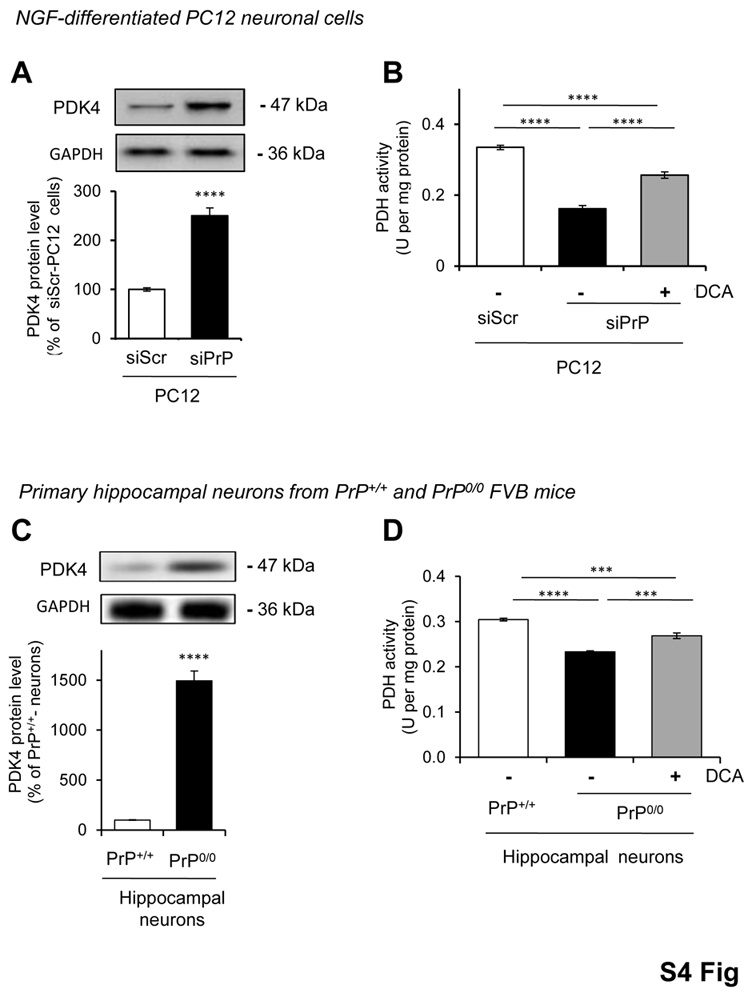

Supplement: S4 Fig — (A) Representative western blot and quantification histogram of PDK4 protein expression level and (B) PDH activity in PC12 neuronal cells transiently silenced for PrPC expression (SiPrP) or not (SiScr) (n = 6). PC12 cells were treated with DCA (2 mM) for 6h. (C) Representative western blot and quantification histogram of PDK4 protein expression level and (D) PDH activity in primary cultures of hippocampal neurons isolated from adult FVB and PrP0/0-FVB mice and left to regenerate for 10 days (n = 6). Hippocampal neurons were treated with DCA (2 mM) for 6 h. GAPDH was used for normalization in western-blot experiments. Data are the mean ± SEM. *** denotes p < 0.001 and **** p < 0.0001. (TIF) [file ppat.1009991.s004.tif]

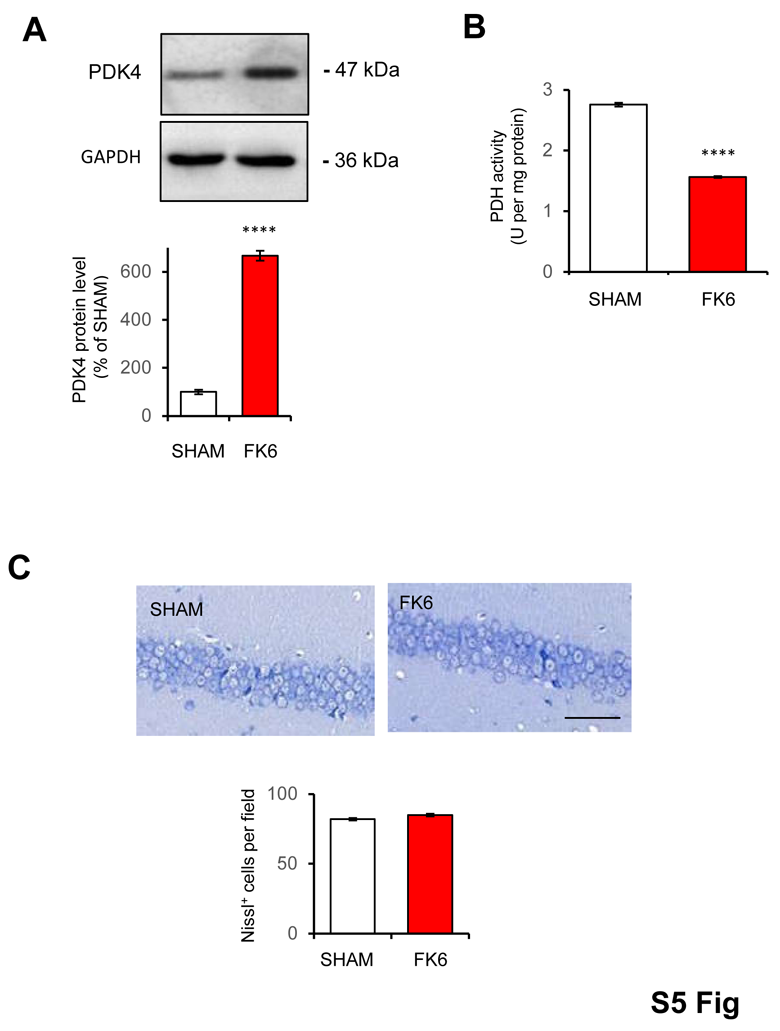

Supplement: S5 Fig — (A) Representative western blot of PDK4 protein expression level, (B) PDH activity, and (C) Nissl staining of viable neurons in the hippocampus of C57Bl6/J mice infected or not with Fk6 prions sacrificed at 130 days post-infection (n = 4). Scale bar = 100 μm. GAPDH was used for normalization in western-blot experiments. Data are the mean ± SEM. **** denotes p < 0.0001. (TIF) [file ppat.1009991.s005.tif]

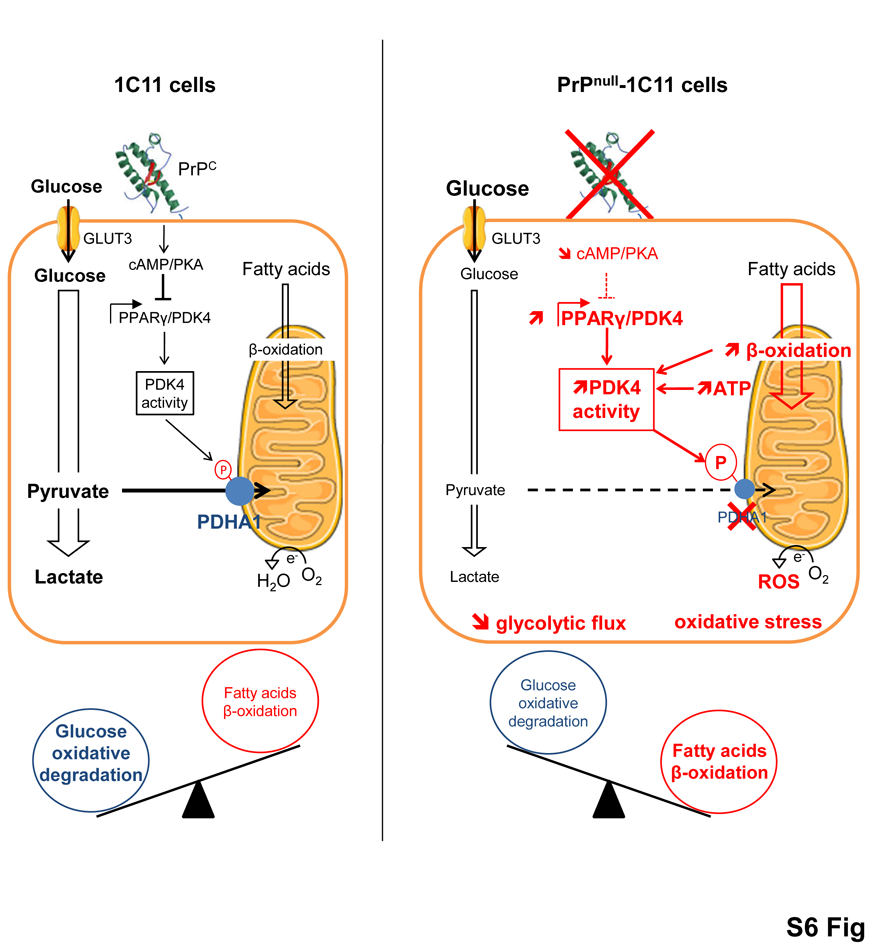

Supplement: S6 Fig — In PrPnull-cells, loss of PrPC coupling to the cAMP/PKA signaling pathway abrogates PrPC negative control of PPARγ and PDK4 expression. The subsequent rise in PDK4 level and activity leads to a reduction of PDH complex activity associated with a rise of phosphorylation of the PDHA1 subunit. This provokes a decrease of the glycolytic flux in favor of an increase in the fatty acids β-oxidation rate, which combined to the excess of synthesized ATP sustain high PDK4 activity. The in fuel preference of PrPnull-cells towards the use of fatty acids is accompanied by the onset of oxidative stress conditions. In PrPC-expressing cells, PrPC coupling to cAMP/PKA signaling tones down PPARγ/PDK4 expressions, which equilibrates carbohydrate and fatty acid degradations, and thereby confers anti-oxidative stress function to PrPC. (TIF) [file ppat.1009991.s006.tif]

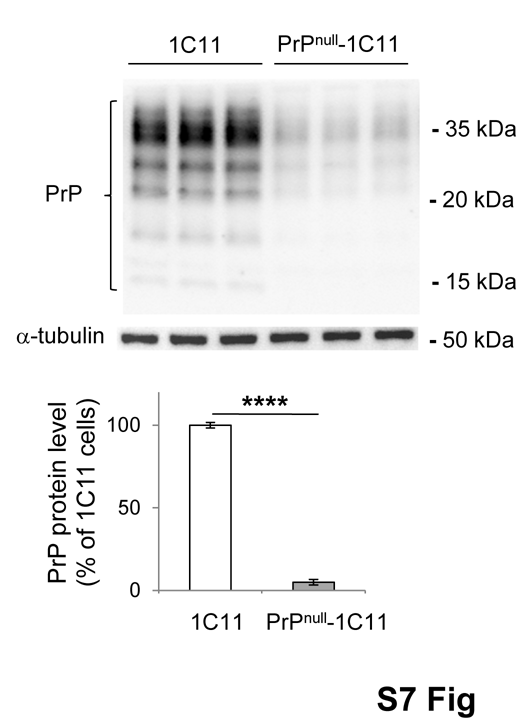

Supplement: S7 Fig — Representative western blot and quantification histogram showing PrPC depletion (>95%) in 1C11 cells chronically silenced for PrPC upon constitutive expression of a siRNA targeting PrP mRNA compared to parental 1C11 cells [31] using Sha31 PrP antibody (n = 3). α-tubulin was used for normalization. Data are the mean ± SEM. **** denotes p < 0.0001. (TIF) [file ppat.1009991.s007.tif]
