# Supplementary material for: Loss of prion protein control of glucose metabolism promotes neurodegeneration in model of prion diseases
Source: PLoS Pathog. 2021 Oct 5;17(10):e1009991. doi: 10.1371/journal.ppat.1009991 (PMC8519435; doi:10.1371/journal.ppat.1009991)
Supplement: Attachment — Submitted filename: Response to reviewers.pdf [file ppat.1009991.s011.pdf]

Université de Paris

Inserm

Dear Pr. Agrimi,

Please find enclosed herewith a new version of the manuscript entitled "**Loss of prion protein control of glucose metabolism promotes neurodegeneration in model of prion diseases**" by Arnould H, Baudouin V, Baudry A, Ribeiro LW, Ardila-Osorio H, Pietri M, Caradeuc C, Soultawi C, Williams D, Alvarez-Meija M, Crozet C, Djouadi F, Laforge M, Bertho G, Kellermann O, Launay JM, Schmitt-Ulms G, and Schneider B submitted for publication at *PLOS Pathogens*.

This new version takes into account the editorial requests and other concerns raised by the three reviewers. A detailed point-by-point answer is provided below.

We hope that you will find this new version of our manuscript now suitable for publication at *PLOS Pathogens*.

Sincerely yours,

Benoit Schneider, Ph.D.
Benoit.schneider@parisdescartes.fr

Answers to the four editorial concerns:

1. *In vitro experiments are limited to a single cell model. The most important changes in PrP^{null}-1C11 cells (e.g. expression level of PDK4 and PDH activity) should be verified in other relevant PrP^{null}-cells and tissues.*

We now provide additional data showing that transient siRNA-based silencing of PrP^{C} in the PC12 neuronal cell line also triggers a rise in the protein expression level of PDK4 (2.5-fold) and a decrease of mitochondria PDH activity (50%) compared to PC12 cells transfected with an unrelated siRNA.

As for PrP^{null}-1C11 cells, the inhibition of PDK4 with dichloroacetate (DCA, 2 mM) partly rescues PDH activity in PrP^{C}-silenced PC12 neuronal cells, indicating that PDK4 overexpression caused by PrP^{C} downregulation contributes to PDH activity deficit.

These new data obtained with PC12 neuronal cells again support the functional implication of PrP^{C} in the regulation of glucose metabolism through control of the PDK4-PDH axis. These results are now introduced as **new S4 Fig.** in the improved version of the manuscript (page 12).

2. *Immunoblot data showing the PrP^{C} expression levels of PrP^{null}-1C11 cells should be included which rule out the possibility of some residual PrP expression.*

As described in Loubet et al. (FASEB J, 2012) and Ezpeleta et al. (Scientific Reports, 2017), PrP^{null}-1C11 cells are chronically repressed for PrP^{C} expression by at least 95% compared to parental 1C11 cells. As requested, we introduced western-blot experiments (**new S7 Fig.**) confirming PrP^{C} down-regulation by >95% in PrP^{null}-1C11 cells using Sha31 PrP-targeting antibody. This is also clearly indicated in the introduction section (page 5).

Université de Paris   Inserm

3. *In vivo studies are done in prion-infected mice at terminal stage of disease when many different cellular functions are likely to be affected. To avoid confounding effects of metabolic changes downstream of neurodegeneration, it would be important to confirm results in prion-infected mice at earlier disease stage, and test whether DCA treatment alleviates incipient neurodegeneration.*

In this improved version of the manuscript, we quantified at the end-stage of the disease Nissl-stained viable neurons in the hippocampus of prion-infected mice infused or not with dichloroacetate (DCA). We show that prion infection provokes a 50% decrease of hippocampal neurons compared to non-infected mice. Upon PDK4 inhibition with DCA, the neuronal cell population in the hippocampus of prion-infected mice was reduced by 30% as compared to SHAM mice. As the level of PrP^{Sc} in the hippocampus did not differ between prion-infected mice infused or not with DCA (**Fig. 6I**), these new data support the view that counteracting PrP^{Sc}-induced metabolic abnormalities on PDK4 inhibition protects, at least partly, neurons from degeneration, and thereby extends the survival time of prion-infected mice (**Fig. 6A**). These results are now introduced in the result section and **figure 6 as new panel 6J**. (page 17).

In addition, we measured PDK4 expression level, PDH activity, and Nissl-stained viable neurons in the hippocampus of prion-infected mice at an early stage, *i.e.*, at 130 days post-infection, that is, 10 days before the onset of clinical signs, *versus* uninfected mice. We monitored a rise in PDK4 expression and a deficit of PDH activity but no significant changes in the population of viable neurons in the hippocampus of prion-infected mice at 130 dpi. This indicates that PrP^{Sc}-induced metabolic changes precede incipient neurodegeneration. These additional data were inserted in the result section as **new S5 fig.** (page 17).

4. *An experiment that would strengthen the in vivo relevance would be the reduction of PDK4 in brain homogenates of prion-infected mice, tested in immunoblot (and reversion in inhibitor-treated mice).*

The data in Figure 6 support the view of a loss-of-PrP^{C} regulatory function toward glucose metabolism within a prion-infectious context. We rather expect an increase in PDK4 expression level in the brain of prion-infected mice than a reduction. We now show in the hippocampus of prion-infected mice at the end stage of the disease a rise in PDK4 protein level (3.5-fold) as compared to non-infected mice (immunoblot; **new Fig. 6B**). DCA infusion in prion-infected mice reduced PDK4 protein level, likely reflecting repression of PDK4 expression caused by the reduction of fatty acids β-oxidation as described in Jeong et al. (2012) in favor of glucose metabolism.

Jeong JY, Jeoung NH, Park K-G, Lee I-K. Transcriptional regulation of pyruvate dehydrogenase kinase. Diabetes Metab J. 2012;36: 328–335. doi:10.4093/dmj.2012.36.5.328

Answers to other concerns raised by the three reviewers:

i) We now justify in the result section the rationale of focusing our metabolic study on the hippocampus of prion-infected mice at the end-stage of the disease (page 16). (Reviewers 1&3).

ii) The reference Arima et al. (2005) that recapitulates the tropism and neuropathology properties of Fukuoka prion strain was introduced in the manuscript. (Reviewer 3).

Arima K, Nishida N, Sakaguchi S, Shigematsu K, Atarashi R, Yamaguchi N, et al. Biological and biochemical characteristics of prion strains conserved in persistently infected cell cultures. J Virol. 2005;79: 7104–7112.

iii) Supplementary table 1 was improved and now mentions several parameters associated with the MS characterization of proteins expressed differentially between PrP^{null}-1C11 cells and PrP^{C}-expressing cells (number of peptides, sequence coverage…). (Reviewer 1).

iv) Based on the fact that DCA is a drug approved for treating lactic acidosis and some solid tumors and DCA exerts a beneficial effect towards prion disease in mice, we now discuss the use of DCA as a potential disease-modifier drug for the treatment of Creutzfeldt-Jakob disease patients (page 23). (Reviewer 1).

v) We now introduce the suggested references (Gasperini et al., 2015; Slpasak et al., 2016) in the introduction section. (Reviewer 2).

vi) We now justify in the discussion section the rationale of treating prion-infected mice at a late stage of the disease, *i.e.*, at 130 dpi, that is, 10 days before the onset of clinical signs (page 23). (Reviewer 2).

vii) We now provide evidence for the impact of metabolic changes on neurodegeneration in prion-infected mice (please see above the response to point 3). This validates the proposed title as we show metabolic changes precede incipient neurodegeneration according to Nissl staining of viable neurons in the hippocampus of prion-infected mice (Reviewers 1&3)

viii) Representative immunoblots of the protein changes shown in Fig. 1C were introduced as new S2 Fig. (Reviewer 3).

ix) As rightly outlined by reviewer 3, the y-axis in Fig. 6A represents the percentage of mice survival, *i.e.*, the time to terminal disease. This was changed accordingly.
