# Supplementary material for: Loss of prion protein control of glucose metabolism promotes neurodegeneration in model of prion diseases
Source: PLoS Pathog. 2021 Oct 5;17(10):e1009991. doi: 10.1371/journal.ppat.1009991 (PMC8519435; doi:10.1371/journal.ppat.1009991)
Supplement: Attachment — Submitted filename: Response to reviewers.docx [file ppat.1009991.s012.docx]

We deeply thank reviewers 1 and 2 for their nice appreciation of our work and of the changes that we made according to their suggestions and comments.

**Reviewer #3**

1- The authors do not reconcile the findings in 1C11 cells with transcriptomic data that are available from Zurich-3 PrP KO mice. These data are reported in Nuvolone et al. (2016), which is referenced within the manuscript. Examination of these data shows that the relevant genes not dysregulated in the Zurich-3 mice. This discrepancy seems to undermine the conclusions of the paper. The authors suggest in the Introduction the general argument that embryonic compensatory mechanisms may play a role in the lack of phenotype of PrP KO mice, but they do undertake a detailed discussion of those transcripts that are altered in the Zurich 3 mice.

We find the concern raised by reviewer 3 out of the scope of the present study, as there is no rationale for undertaking a focused discussion of the transcripts altered specifically in Zurich-3 (ZH3) PrP KO mice. Our work relies on a proteomic approach that compares the proteome of 1C11 cells silenced for PrP^C^ to that of PrP^C^ expressing cells.

Nevertheless, according to Nuvolone et al. (2016), only 14 genes (mainly connected to immune functions) are impacted in the hippocampus of ZH3 PrP KO mice *vs.* C57Bl/6J mice. However, in a current preprint study by Matamoros-Angles A. et al. (2021), RNA seq experiments performed in the hippocampus of those ZH3 PrP KO mice *vs.* C57Bl/6J mice reveal that many other genes (whose list is not presented) are also differentially expressed upon the *Prnp* genetic ablation. These genes relate to the control of neuronal functions (neurotransmitter receptors, synapse proteins) and energy metabolism. Compared to the leading publication by Nuvolone et al. (2016), the study by Matamoros-Angles et al. thus deeply extends the repertoire of genes and cell functions impacted by PrP^C^ ablation in ZH3 PrP KO mice, complexifying the analysis of the phenotype of ZH3 PrP KO mice.

At a functional level, Matamoros-Angles et al. also report a deficit in neuronal maturation, network formation, and neuronal excitability in *Prnp*^ZH3/ZH3^ neuronal cultures. This study supports many works done by others and corroborates past works from our laboratory on PrP^C^ function, *i.e.*, the implication in neuritogenesis. As the paper by Matamoros-Angles A. et al. is still a preprint article, it cannot, unfortunately, be cited in our manuscript.

Reference.

A. Matamoros-Angles, A. Hervera, J. Soriano, E. Martí, P. Carulla, F. Llorens, M. Nuvolone, A. Aguzzi, I. Ferrer, A. Gruart, JM. Delgado-García, JA. Del Río. Behavioral deficits, learning impairment, and enhanced hippocampal excitability in co-isogenic PrnpZH3/ZH3 mice. doi: <https://doi.org/10.1101/2021.02.20.432083> (preprint).

2- The use of shRNA (1C11 cells) or siRNA (PC12 cells) are not the most up-to-date or effective ways to eliminate gene expression. The authors should use CRISPR-Cas to completely ablate expression of PrP (either via introduction of indels or by excision of the gene). The metabolic changes should then be reversible by re-introduction of PrP expression. In addition, the concern about clonal variability of the cell lines (raised by a previous reviewer) is not adequately addressed. Both 1C11 and PC12 are transformed cells lines with abnormal and unstable ploidy, so clonal variability is a serious limitation. The authors would be better off using primary neurons from PrP KO mice.

We have performed additional experiments with primary hippocampal neurons isolated from adult FVB mice KO for PrP^C^ expression (PrP^0/0^-FVB mice) that show overexpression of PDK4 and reduction in PDH activity as compared to hippocampal neurons isolated from wild-type FVB mice. The inhibition of PDK4 with DCA increases, at least partly, PDH activity in primary PrP^0/0^-hippocampal neurons (new supplementary Figures S4C and S4D). These new experiments validate our data obtained with 1C11 and PC12 cells silenced for PrP^C^ expression.

3- As pointed out by the previous reviewers, the data from prion-infected mice may reflect non-specific metabolic alterations secondary to prion infection, rather a specific loss of PrP function. This concern is still not adequately addressed. In this regard, the title (which does not appear to have been changed) and overall conclusion that “Loss of prion protein control of glucose metabolism promotes neurodegeneration in a model of prion diseases” in unjustified. A long-standing question in the field has been whether prion diseases are due to a loss-of-function of PrPC or a gain-of-function of PrPSc. Animals devoid of PrP display, at most, minimal phenotypic abnormalities, and certainly not any features of a prion disease. In addition, there is evidence that significant levels of PrPC are present even at end stage of disease (Mays et al., 2015 doi:10.1128/JVI.02142-15). These findings argue strongly against loss of PrPC function a major contribution to the phenotype of prion diseases. This conclusion, which is diametrically opposed to the title of the paper, remains a fundamental problem with this study.

At the plasma membrane, PrP^C^ belongs to multiprotein complexes and acts as a signaling molecule or a scaffolding protein regulating the assembly and activity of diverse signaling modules. Within a prion infectious context, the exclusion of PrP^C^ from those multiprotein complexes and/or the modification of the stoichiometric interaction between PrP^C^ and its partners upon PrP^Sc^ interaction with PrP^C^ would cancel PrP^C^ regulatory roles in neurons, *i.e.*, induce a loss-of-PrP^C^ function, without depleting PrP^C^. The rise in PrP^C^ level observed at the end stage of prion diseases could be a defense mechanism trying to restore lost PrP^C^ protective functions.

Our experimental approach consisting in downregulating PrP^C^ expression is one way to grasp the concept of loss-of-PrP^C^ function. In addition, depending on the readouts, the fact that PrP^C^-depleted systems (PrP^null^-cells as well as PrP^0/0^ mice) phenocopy prion-infected paradigms argues for a contribution of a loss-of-PrP^C^ function(s) in prion diseases (see Pietri et al. Nat. Med., 2013, Alleaume-Butaux et al. Plos Pathogens, 2015, and Ezpeleta et al. Scientific Reports, 2017).

Here, by showing overexpression of PDK4, a deficit of PDH activity, and reduction of glycolytic flux in both PrP^null^/PrP^0/0^ cell systems and the hippocampus of prion-infected mice, our data support the view of a loss-of-PrP^C^ function toward the regulation of glucose metabolism within a prion-infected context. Moreover, we added in our previous version of the manuscript experiments showing that metabolic abnormalities precede neurodegeneration (see supplementary Figure S5) and the inhibition of PDK4 with DCA (that rescues oxidative degradation of glucose) protects, at least partly, neurons from degeneration (see Figure 6) in the hippocampus of prion-infected mice. These data indicate that PrP^Sc^-induced metabolic derangements contribute to neurodegeneration.

For all these reasons, we deem that the title and the concept conveyed in our study are fully justified and supported by our experiments.

References.

Pietri M, Dakowski C, Hannaoui S, Alleaume-Butaux A, Hernandez-Rapp J, Ragagnin A, Mouillet-Richard S, Haik S, Bailly Y, Peyrin JM, Launay JM, Kellermann O, Schneider B.PDK1 decreases TACE-mediated α-secretase activity and promotes disease progression in prion and Alzheimer's diseases. Nat Med. 2013 Sep;19(9):1124-31. doi: 10.1038/nm.3302. Epub 2013 Aug 18.

Alleaume-Butaux A, Nicot S, Pietri M, Baudry A, Dakowski C, Tixador P, Ardila-Osorio H, Haeberlé AM, Bailly Y, Peyrin JM, Launay JM, Kellermann O, Schneider B. Double-Edge Sword of Sustained ROCK Activation in Prion Diseases through Neuritogenesis Defects and Prion Accumulation. PLoS Pathog. 2015 Aug 4;11(8):e1005073. doi: 10.1371/journal.ppat.1005073. eCollection 2015 Aug.

Ezpeleta J, Boudet-Devaud F, Pietri M, Baudry A, Baudouin V, Alleaume-Butaux A, Dagoneau N, Kellermann O, Launay JM, Schneider B. Protective role of cellular prion protein against TNFα-mediated inflammation through TACE α-secretase. Sci Rep. 2017 Aug 9;7(1):7671. doi: 10.1038/s41598-017-08110-x.

**Minor issues**

Reviewer #2: My only suggestion would be to tone down the final outcome of the study. In fact, to my knowledge, DCA is not approved by the FDA as a cancer treatment, and its current use is exclusively for congenital lactic acidosis. More generally, the therapeutic benefits of DCA in lactic acidosis, cancer, and post-ischemic heart recovery have been quite controversial. I recommend the authors to explain in a more critical perspective the known limits and the possible side effects (e.g., peripheral neuropathy) of DCA in the clinical practice, also in light of the limited effects reported in this study.

As rightly outlined by reviewer 2, DCA is not formally approved by the FDA as a cancer treatment even if several studies demonstrated beneficial effects of PDK4 inhibition in this last context. We thus modify the sentence “Face today’s absence of any medicine to combat prion diseases, our data thus suggest dichloroacetate, a drug already approved for the treatment of lactic acidosis and several different solid tumors [76,77], would be repurposed as a symptom-modifying drug for treating Creutzfeldt-Jakob disease patients.” by “Face today’s absence of any medicine to combat prion diseases, our data thus suggest dichloroacetate, a drug already tested in diverse clinical trials for the treatment of congenital lactic acidosis, and several solid cancers [76, 77], would be used as a symptom-modifying drug for treating Creutzfeldt-Jakob disease patients.” (page 23 in the discussion section).

Of note, the main messages conveyed by our study are (i) the identification of the energetic metabolism as a novel cell function controlled by PrP^C^, and (ii) the deregulation of this PrP^C^ metabolic role within a prion-infected context and contribution to neurodegeneration. As DCA was notably used as a pharmacological tool to delineate the contribution of PDK4 overactivity to prion diseases, we consider discussing the known limits and the possible side effects of DCA in the clinical practice fells under the scope of this paper.
